# Attitude of nursing students following the implementation of comprehensive computer-based nursing process in medical surgical internship: a quasi-experimental study

**DOI:** 10.1186/s12911-020-01378-6

**Published:** 2021-01-06

**Authors:** Kobra Parvan, Fahimeh Alsadat Hosseini, Madineh Jasemi, Brian Thomson

**Affiliations:** 1 Medical Education Research Center, Health Management and Safety Promotion Research Institute, Tabriz University of Medical Science, Tabriz, Iran; 2 Department of Medical-Surgical Nursing, School of Nursing and Midwifery, Fasa University of Medical Sciences, Ibn Sina Square, Fasa, Fars Province Iran; 3 Department of Medical-Surgical Nursing, School of Nursing and Midwifery, Urmia University of Medical Sciences, Urmia, Iran; 4 School of Health and Life Sciences, University of the West of Scotland, Paisley, Scotland

**Keywords:** Attitude, Nursing student, Software, Nursing process

## Abstract

**Background:**

The nursing process is the core and the standard of practice in nursing profession. Nowadays, the use of information technology in the field of nursing processes, education and practice has been emphasized. Since nurse’s attitudes towards clinical information systems are considered as an indicator of the success rate of information systems, and nurse’s attitudes about the nursing process can affect their execution of the process. So the purpose of this study was to evaluate nursing students’ attitudes towards the nursing process software.

**Methods:**

In this quasi-experimental study, 160 undergraduate nursing students (terms 4–8) in Tabriz University of Medical Sciences were selected by convenience sampling. To evaluate the effectiveness of nursing process software in this study, Mazlom and Rajabpoor (IJME 14(4):312–322, 2014) a questionnaire consisting of 21 components based on a five-point Likert scale was completed by students after using the software. Data were then analyzed by SPSS 19 software.

**Results:**

The mean score of students’ attitude toward nursing process software was high (80.70 ± 5.58). The nursing students’ highest scoring attitudes were respectively related to “Effectiveness of software in prioritizing patient care and problems”, “Completeness of patient’s electronic information compared to handwritten mode” and “Software’s effectiveness in saving your time”. The lowest scoring attitudes towards the software was respectively related to the “feeling of fairness in labor division”, “the effectiveness of the software in determining your workload” and “the feeling of satisfaction in labor division”. There was a statistically significant relationship between gender and age, and student’s attitude toward nursing process software.

**Conclusions:**

According to the results and analysis of nursing student’s attitudes toward nursing process software, the use of such software would be welcomed by students. It seems that changing policies in the educational and clinical substructure of nursing in order to develop, adapt and use the nursing process software is an important responsibility for nursing authorities to consider. Providing educational and clinical technology equipment, periodic evaluation of software by stakeholders and promoting the use of this software, can be fundamental steps in operationalizing the findings of this research.

## Background

The nursing process is a systematic and dynamic method of patient care [1]. The nursing process is at the core [2] and standard practice in the nursing profession [3]. The use of the nursing process has contributed to the delivery and planning of desirable, clear and effective nursing care, and is ultimately effective in improving the quality of patient care [4].

Based on some studies, the nursing process faces many challenges in practice [5]. A systematic approach to teaching and implementing the nursing process is rarely followed in hospitals [2]. Mamseri [6] found in his study that 81% of nurses are trained in the nursing process, but only 43% are able to perform it and according to the results of one study the majority of nurses use the nursing process in their nursing care incompletely [7, 8]. Evidence shows that not only is the nursing process incompletely performed by nurses working in hospitals, but also by nursing students and teachers [9–11].

Not applying the nursing process as standard in care causes problems such as decreased job satisfaction, decreased scientific nursing practice, thoughtless obedience, reduced quality of care delivery and over dependence on physicians [10].

Factors affecting the implementation of the nursing process are varied and complex [12]. One of the barriers to the implementation of the nursing process is the ‘low value’ attitude of nurses towards the nursing process [3]. Nurses who have a positive knowledge and attitude towards the nursing process are more interested in using it [13]. According to some experts, the nursing process in the current context is unclear, time consuming and difficult to implement [14].

Based on some studies, there have been problems in establishing and using the nursing process in many nursing institutions, especially in developed countries [15]. These studies identified the educational environment, together with the characteristics of nursing in Iran as ‘ambiguous’ in routine care. Other problems identified have been; dependent and uncritical thinking [16], lack of clinical facilities and equipment, lack of a consistent and systematic curriculum for teaching and evaluating the nursing students [17], together with limited educational opportunities [18]. This indicates the need to reform the educational curriculum and infrastructure of nursing in Iran. In the United States, all nursing students are trained using the nursing process within the first year of entering the nursing field [19]. The nursing education focus of the United States is on developing critical thinking skills in nursing students enabling them to deliver comprehensive patient-centered care to a variety clients and conditions, and encouraging the use of evidence-based practice in a culturally aware and safe manner [20]. Some nurse education challenges identified in the USA are: meeting nursing shortages, removing confusing and inconsistent educational materials, providing great clinical educational experiences for students and being willing to engage in designing innovative methods for educating nurses in the future [21]. Using a comprehensive computer-based nursing process, as an innovative educational method may help in meeting these challenges [22, 23].

Therefore, it seems necessary to find other methods to improve nursing students’ attitudes towards the nursing process and persuade them to practice this process in clinical settings in the future [10]. Nursing instructors should develop new learning methods to prevent superficial learning, and develop their critical thinking skills, problem solving and also their knowledge and information retention [24]. Improving and developing different learning methods and integrating models are important activities that have been addressed in recent years [25]. In this regard, it should be noted that new and interactive learning methods based on technology are recommended as they have many advantages over traditional methods of nurse education [22]. Information technology can use to improve clinical documentation and support the development of computerized nursing process software. This would help to integrate a logical structure of data, information and knowledge when making nursing care decisions [26]. Professionals should use these technologies and integrate training in computer science into nursing education and thus enhance the nursing process [23]. Advances in nursing informatics also aim to increase the time available for professionals to perform humanitarian and care activities [27].

Many methods have been used to evaluate the consequences of implementing information systems [28]. Among these methods, surveying information systems users’ attitudes is still considered as an effective and efficient way of obtaining feedback from nurses about the effectiveness of information systems [29]. Studies have shown that nurses’ ‘attitudes and satisfaction’ with clinical information systems are considered as a predictor of the success rate of the use of information systems [30–32] and nurses’ attitudes towards the nursing process can affect the implementation of the process [19]. These educational challenges in clinical nursing [16–21], undeniably benefit from the use of the nursing process [33] and its application in clinical care [9]. This paper explores the attitudes of student nurses towards the use of software to enhance and encourage the implementation of the nursing process. Together with the importance of using information technology in nurse teaching and practice [26]. The purpose of this study was to evaluate attitudes of nursing students towards using nursing process software.

### Study hypothesis

Considering various merits of utilizing the nursing process [33] and especially incorporating an information technology approach [23] and given the positive response towards the use of nursing process software and its ability to enhance evidence based practice, this study proposes the following hypothesis:

H1: using computer-based nursing process software to support patient care in medical/surgical internship courses, positively influences the attitude of undergraduate nursing students toward utilizing the computer-based nursing process software when delivering patient care.

### Related work

Table 1 shows the latest research related to using computer based programs in nursing.

**Table 1:** The latest studies about development and using the computer based programs in nursing

Author/year publication/country	Objective	Findings	The study characteristics
BabaMohamadi et al. (2013) [28]/Iran	Assess nurses’ attitude toward the effect of nursing electronic reports on patient care	Poor nurses’ attitudes about the nursing electronic reports	Design/ sampling method: descriptive-analytical study/ Census sampling the study sample: 316 nurses The feature of the software: evaluating nursing electronic reports
Sayadi and Rokhafroz (2013) [10]/Iran	Determining nursing students’ perspectives about a mobile software on nursing process for bedside use	Improving knowledge and skills of most students about nursing process after using the software	Design/sampling method: pre experimental one group design/convenience sampling the study sample: 30 Nursing Students The feature of the software: developing the mobile software according to the learning objectives regarding nursing process in cardiology ward (including all stages of nursing process)
Mazlom and Rajabpoor (2014) [34]/Iran	Design and assess the local nursing process computerized software	software development feasibility according to health system of Iran and satisfaction of nurses and students with software	Design and assess the software The study sample: 10 nursing students (in the last semester) and 10 nurses in ICU ward The feature of the software: This pilot software included all nursing process stages
Topaz et al. study (2017)[35]/Finland	Qualitative content analysis of nurses’ satisfaction and issues with current electronic health record (EHR) systems	Low satisfaction rankings of the most participants about electronic health record (EHR) systems	Design/sampling method: cross-sectional survey design/snowball sampling strategy/the study sample: 469 nurses The feature of the software: electronic record of nursing care
Hariyati et al. (2018)[36]/Indonesia	Exploring nurse satisfaction of the new computer-based information system	Improvement on satisfaction of simplicity and completeness of nursing process after using computer-based nursing information system	Design/sampling method: cross-sectional approach, pre and posttest design/purposive sampling The study sample: 27 nurses The feature of the software: nursing documentation
Lima, Vieira and Nunes (2018)[37]/Paraiba	Development of software to support decision-making for the selection of nursing diagnoses and interventions for children and adolescents	Contributing of the tool in decision-making and quality of care	Design and development of the software The feature of the software: The software processes some parts of nursing process including nursing diagnoses, outcomes and interventions of a university hospital

It should be noted that in most nursing computerized programs used in the studies above addressing just some nursing tasks or some nursing process steps appears to be the case, and in the majority of these studies, the attitude of nursing students wasn’t assessed. Also these studies weren’t based on an educational approach. Therefore, this study used software that included all stages of the nursing process. Then assessed the attitude of nursing students toward this comprehensive computer-based nursing process software in medical/surgical internships.

## Methods

This study is a quasi-experimental study that was done to determine the attitudes of nursing students regarding the nursing process software. We used the nursing students of Tabriz University of Medical Sciences in 2018. The study population consisted of all undergraduate nursing students in terms 4–8. The total number in our sample who met the inclusion criteria was 160. People were selected by the convenience sampling method. Criteria for participation in the study included; willingness to participate in all stages of study, undergraduate terms 4–8 students at Tabriz University of Medical Sciences, basic knowledge of computer use, and 2 weeks of continuous use of the nursing process software (with a minimum of 4 patients).

In this study, a two-part questionnaire was used. The first part consisted of socio-demographic characteristics of the participants and consisted of 11 questions. And the second part was Mazlom and Rajabpoor’s [34] questionnaire which assessed nursing students’ attitudes towards nursing process software. This questionnaire consisted of 21 items based on a five-point Likert scale (1: very low, 2: low, 3: average, 4: good, and 5: very good). The validity and reliability of the primary instruments in the study by Mazlom and Rajabpoor [34] were investigated in Iran. The content validity of the questionnaire was confirmed by 10 experts and the reliability of the tool was assessed by retesting method (0.81) [34]. The questionnaire’s score is calculated based on the sum of the total scores.

Ethical approval was granted by the Ethics Committee of Tabriz University of Medical Sciences (IR. TBZMED.REC.1393.214). Then some information about the research and assurance regarding the confidentiality of the information obtained, and the privacy and of the participants was provided. Participants were also assured that participation was optional. After containing written consent from participants and explaining goals of the study, undergraduate nursing students from terms 4–8 of Tabriz University of Medical Sciences who were undergoing internship in medical/surgical wards were selected using convenience sampling method.

‘Nursing process software’ is a computer based program that was developed using a systematic and scientific problem solving approach to help identify and treat patient problems using a series of organized steps (Assessment, nursing diagnosis, Goals and expected outcomes, Planning, Implementation and Evaluation.) The software is designed to provide a platform for the efficient and effective provision of nursing care.

To design the nursing process software, extensive text reviews and the latest international standards relating to the American nursing association, NANDA were consulted. In addition, the nursing diagnosis list (2018–2020) and also reference books in Nursing such as Ulrich and …. were used. The software consisted of six steps; 1. Assessment 2. Nursing diagnosis 3. Determining goals and expected outcomes 4. Planning 5. Implementation and 6. Evaluation. The software was designed in CD and web-based forms. This way, students could use this software through their cell phones using their network connection.

At first, after describing the purpose and nature of the study to the participants, the nursing process software was installed on students’ smartphones. Initially the students were taught how to use the software, then to increase the students understanding, software performance results from a patient were shown to the students and students’ questions and ambiguities about the software were answered. Nursing process software was provided to students for 2 weeks working with patients. Patients are assigned to students once every 3 days and they have to report the patient’s condition in a clinical meeting at the end of the third day (The report includes a description of patients’ assessment and diagnosis and then the expected nursing goals and outcomes, then nursing care plans, implementation and performed evaluations undertaken while using the software), this report is given to their clinical instructor and other students and within the presence of the researcher. It should be noted that the nursing interventions provided to the patient by the student are documented in the software. On the last day of the intervention, nursing students delivered a final copy of the nursing process software covering all the stages of patient care and evaluation to the researcher.

Each student enters his/her account with a username and password. Then students should enter the patients’ data obtained from the patient assessment and examination in the patient assessment section of the software. Then they can access their patients nursing diagnoses by clicking on next step tab, and then by clicking again on next step tab they are able to progress through the software to information about the expected goals and outcomes of the patient. This process continues until the last stage of the nursing process that is the evaluation stage, and these steps can be repeated according to the care goals of patients that are identified in the evaluation stage. In addition, it is possible for students to access different parts of the nursing process non-consecutively according to their needs. Students can edit the patient’s data at any time and this data can be stored and retrieved by the student.

In the evaluation phase, students are able to access all patient problems. At this stage, their success rate in solving problems is indicated by a percentage. In addition, other problems that have not yet been resolved are shown for further consideration. Also, the option of restarting the nursing process due to unresolved problems is possible. In this way, the student can have feedback on his care performance and the patient’s condition by using the nursing process software.

It is intended that the features of this software, include: being user friendly, addressing the patient’s needs comprehensively, fast processing, and providing evidenced and up-to-date care content, without the need to refer to various nursing resources. The software guides the students from the initial assessment through to the final evaluation, as well as offering electronic documentation, eliminating the need to provide time consuming documentation. The software provides an innovative learning tool for providing effective patient-centered care and so can play an important role in creating a positive attitude among students using this software in the patient care process. So, after using the software, the nursing students in medical surgical internship course, completed the attitude-test questionnaire about nursing process software using self-report method. The data were collected from May to June of 2019.

**Fig. 1:**
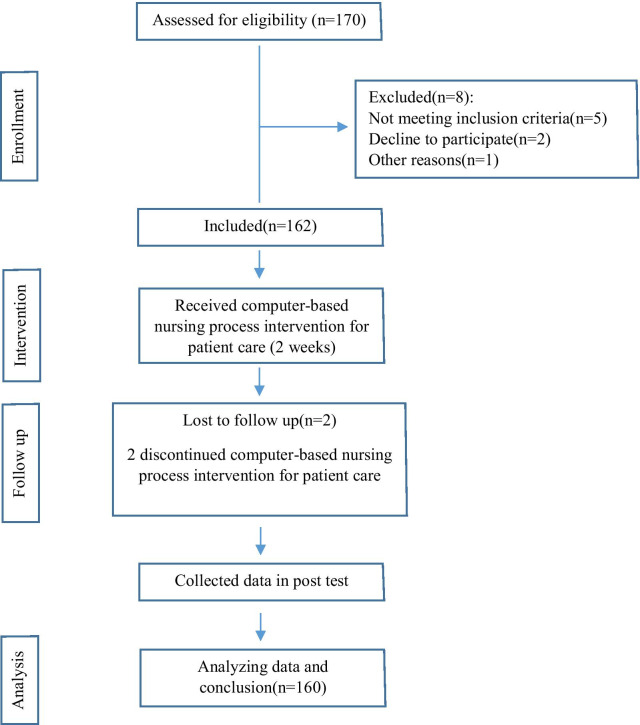
The flow diagram of the study methodology

After data collection, data were analyzed by SPSS (Statistical Package for Social Sciences) software version 19 using descriptive statistics test (mean and standard deviation) and analytical statistics (independent *t* test and one-way ANOVA). Figure 1 shows the flow diagram of the study methodology.

### Limitations of the study

Randomization, is not used in this quasi- experimental study. The small population and limited setting of this research study were the main considerations in selecting convenience sampling method.

Another limitation of the study was the lack of a comparison/control group. Due to using one-group only as a quasi- experimental research design. Because, the purpose of this study was to evaluate nursing students’ attitudes about particular computer-based nursing process software, the study questionnaire that was specifically developed to effectively evaluate the student’s attitudes towards that software following a period of using it in their practice would appear reasonable.

The study participants were limited to undergraduate students undergoing a medical/surgical internship course, so it is suggested that this study be repeated among students undergoing other internship courses, and also nurses from a wider variety of wards.

Furthermore, developing a wider range of participants would enable an increase in the intervention period. Since 2 weeks is the common period of internship of each clinical placement evaluating a longer period would increase the validity of the study results.

## Results

In the present study, the total number of participants was 160 people. The mean age of the participants was 23.02 ± 3.13. Also the mean score of students was 16.1 ± 36.18. Nursing interest based on the 10-point scale in the experimental group was 5.19 ± 1.65. Other socio-demographic characteristics of the students participating in the study are presented in Table 2.

**Table 2:** Socio-demographic characteristics of the participants

Socio-demographic characteristics	Frequency (%)
Sex F (%)^a^	
Female	96 (60)
Male	64 (40)
Marital status F (%)	
Single	115 (71.9)
Married	45 (28.1)
Semester F (%)	
Four	27 (16.9)
Five	39 (24.4)
Six	41 (25.6)
Seven	24 (15)
Eight	29 (18.1)
Acquaintance with computer or related software use F (%)	
Yes	153 (95.6)
No	7 (4.4)
Attitude toward computer use in nursing affairs	
Completely agree	46 (28.8)
Agree	68 (42.5)
No ideas	27 (16.9)
Disagree	19 (11.9)
Completely disagree	0 (0)
Nationality F (%)	
Turk	26 (16.3)
Fars	84 (52.2)
Kurd	19 (11.9)
Lor	31 (19.4)
Student work record F (%)	
Yes	63 (39.4)
No	97 (60.6)

^a^Frequency(Percent)

Table 3 presents the mean scores of nursing students based on the Likert scale and frequency (percentage) in each item. In assessing the attitude of students towards the nursing process software, the majority of students scored 16 items out of 21 as good or very good. The nursing students’ highest scoring attitudes were respectively. “Effectiveness of software in prioritizing patient care and problems”, “Completeness of patient’s electronic information compared to handwritten mode” and “Software’s effectiveness in saving your time”. Also, the majority of nursing students did not evaluate any of these items as “weak” or “very weak”. Among the 21 items, those ranked lowest were respectively “feeling of fairness in labor division based on the software”, “the effectiveness of the software in determining your workload” and “the feeling of satisfaction in labor division based on the software” and most students had a moderate attitude in these items.

**Table 3:** Frequency distribution (percentage) of nursing students' attitudes toward the nursing process software and mean and standard deviation of questionnaire's items based on Likert scale

Item	Very low F (%)^a^	Low F (%)	Average F (%)	Good F (%)	Very good F (%)	Mean ± SD^b^
Simplicity of software use	1 (0.6)	1 (0.6)	38 (23.8)	75 (46.9)	45 (28.1)	4.01 ± 0.78
Software installing	8 (5)	22 (13.8)	37 (23.1)	72 (45)	21 (13.1)	3.48 ± 1.05
Simplicity and beauty of software environment	1 (0.6)	8 (5)	52 (32.5)	67 (41.9)	32 (20)	3.76 ± 0.85
Suitable software execution speed	–	9 (5.6)	60 (37.5)	67 (41.9)	24 (15)	3.66 ± 0.8
Printable cares and diagnosis	–	17 (10.6)	47 (29.4)	68 (42.5)	28 (17.5)	3.67 ± 0.89
Effectiveness of software in quality of care improvement	–	14 (8.8)	53 (33.1)	50 (31.3)	43 (26.9)	3.76 ± 0.99
Effectiveness of software in increasing patient’s quality of care	–	2 (1.3)	40 (25)	67 (41.9)	51 (31.9)	4.04 ± 0.79
Effectiveness of software in increasing quality of care cohesion	–	3 (1.9)	22 (13.8)	67 (41.9)	68 (42.5)	4.25 ± 0.76
Effectiveness of software in saving your time	–	3 (1.9)	13 (8.1)	65 (40.6)	79 (49.4)	4.37 ± 0.72
Patient's electronic information’s preciseness compared to handwritten mode	–	1 (0.6)	33 (20.6)	62 (38.8)	64 (40)	4.18 ± 0.78
Accuracy of electronic information compared to handwritten mode	–	–	24 (15)	60 (37.5)	76 (47.5)	4.33 ± 0.72
Completeness of patient's electronic information compared to handwritten mode	–	2 (1.3)	16 (10)	56 (35)	86 (53.8)	4.41 ± 0.72
Effectiveness of software in increasing your level of knowledge	4 (2.5)	1 (0.6)	48 (30)	69 (43.1)	38 (23.8)	3.85 ± 0.88
Effectiveness of software in determining your workload	9 (5.6)	29 (18.1)	68 (42.5)	39 (24.4)	15 (9.4)	3.14 ± 1
Feeling of fairness in work division based on software	10 (6.3)	30 (18.8)	77 (48.1)	43 (26.9)	–	2.96 ± 0.84
Feeling of satisfaction in work division based on software	5 (3.1)	26 (16.3)	68 (42.5)	48 (30)	13 (8.1)	3.24 ± 0.93
Effectiveness of software in identifying alarm signs and doing effective reaction	–	17 (10.6)	51 (31.9)	72 (45)	2. (12.5)	3.59 ± 0.84
Effectiveness of software in organizing patient’s problems	–	–	31 (19.4)	64 (40)	65 (40.6)	4.21 ± 0.75
Effectiveness of software in prioritizing patient care and problems	–	–	10 (6.3)	69 (43.1)	81 (50.6)	4.44 ± 0.61
Effectiveness of software in preventing fault and mistake show by you	–	32 (20)	57 (36.6)	52 (32.5)	19 (11.9)	3.36 ± 0.93
Effectiveness of software in your proper and correct decision making	–	8 (5)	30 (18.8)	79 (49.4)	43 (26.9)	3.98 ± 0.81

^a^Mean ± standard deviation

^b^Frequency (percent)

The mean score of students’ attitude toward nursing process software was 80.70 ± 5.58. Based on the three-part score range, scores of lower than one-third were identified (poor) 21–49, middle one-third (medium) 50–77, and higher one-third (desirable) 78–105, so in this study students’ attitude scores were considered desirable.

When examining the relationship between demographic characteristics and the mean scores of students` attitudes, The Mann–Whitney test showed a significant difference between gender and the mean scores of students’ attitude (*P* value = 0.02), where female students had significantly higher attitude scores toward nursing process software than male students. The Pearson correlation test also revealed a significant difference between the mean scores of students' attitude with age (*r* = − 0.18, *P* value = 0.02), so that younger students’ attitude scores were significantly higher. There was no significant relationship between other demographic variables and students’ mean attitude scores (*p* > 0.05).

## Discussion

The purpose of this study was to determine the attitudes of nursing students towards the nursing process software. The mean score of students' attitudes toward the nursing process software was 80.70 ± 5.58. Based on the three-part range, students’ attitude scores were considered desirable. In this study, the majority of students scored good or very good the majority of items evaluating the nursing process software. Also most nursing students did not evaluate any of the 21 items as weak or very weak. Most students had a moderate attitude in 5 or less items evaluating the nursing process software.

Similar to this study, the attitude of health care providers in other studies conducted in the field of comprehensive software of the nursing process (including all stages of the nursing process) was positive, which indicates the efficiency and effectiveness of such software both in the field of learning and care. According to the study by Hariyati et al. [36], nurses had a positive attitude about using Nursing Management Information System at the Public Health Service in Indonesia, and the nurses satisfaction rating in the area of simplicity and completeness of nursing process increased after using Nursing Management Information System. According to some studies, students were satisfied with the use of nursing process software in clinical practice and its efficiency in promoting clinical care [10, 34]. According to the students’ attitudes in the study by Sayadi and Rokhafroz [10], their knowledge and skills about the nursing process improved after using the software, and 86% of the students said their satisfaction from using software was high or very high. Based on the study of Mazlom and rajabpoor [34], 81.3% of the participants rated the nursing process software as good or very good and also efficiency of software in patient care was very high, so that the mean score of nursing students’ opinions was 84.3 ± 5.83 and for nurses was 73.2 ± 1.13 from total score of 100 and the use of the nursing process software was cited as a facilitator of clinical practice.

Based on the literature review, no negative attitudes about comprehensive nursing process software (including all nursing process stages) were found in the studies. Furthermore, in most studies using non-comprehensive software heath care providers reported lower positive attitudes of the efficiency of the software [38–41]. However in some studies, negative attitudes of the participants about nursing information technology programs were identified [28, 35]. The results of the study by BabaMohamadi et al. [28] showed that more than half of the nurses did not understand the benefits or the impact of the nursing computer program on the patient care process and from their point of view the computer program influenced a limited number of patient care processes. According to the findings of this study, computer programs prevalent in the clinical nursing environment didn’t have complete capability because they couldn’t process information in all or most aspects of care [28]. Also, in a study by Topaz et al. [35], they reported low satisfaction and multi-level concerns with electronic health records in Finland. in this study poor system usability, non-integrated systems, lack of standards; and limited functionality, failure to meet nursing clinical needs, non-nursing-specific systems and a lack of user training were among the main concerns of study participants. It should be noted that in these studies, it was the attitude of health care providers towards the efficacy of nursing electronic records that was assessed and there wasn’t an integrated approach to patients’ assessment and related needs that included care planning and nursing intervention documentation. Similarly, in many studies, the nursing process has been defined as a separate pathway that does not cover all stages and the most unconsidered stage was the evaluation stage [23, 26, 42].

The scientific productions analysis designed to identify the characteristics and requirements of similar systems and software in the area of Nursing care evidenced the importance of the development of Nursing assistance systems to support clinical decisions. These systems considered all the stages of Nursing process [43]. Furthermore, though the nursing process has been divided into parts, they do not occur in isolation but are interdependent and recurring [44, 45]. Expert opinion, appears to agree that the core of the Nursing Process-Clinical Decision Support System should be based on the Advanced Nursing Process as it is research-based. Considering the Nursing diagnoses and true linkages between diagnoses, evidence based interventions, and patient outcomes [46]. In other studies, the use of creative learning models in other areas and the use of different and interconnected phases of the nursing process was mentioned [47]. Therefore, in this study the nursing process software design was based on all interrelated stages of the nursing process as recommended in nursing theory text books. So, it seems obvious that nursing students’ attitudes were significantly positive regarding comprehensive and theoretically based- nursing process software.

In this study, the nursing student’s highest rated attitude was respectively related to the “Effectiveness of software in prioritizing patient care and problems”, “Completeness of patient's electronic information compared to handwritten mode” and “Software’s effectiveness in saving your time”.

Consistent with the findings of the current study and based on the study of Mazlom and Rajabpoor [34], the most important benefits of software from 90% of nurses’ attitudes were being effective in prioritizing diagnoses, accuracy of electronic information compared to handwriting, and helping to organize patient’s problems. Also, in this study, the least frequent measure of satisfaction among 10% of nurses and students participating was related to the accuracy of electronic information compared to handwriting and the effectiveness of the software in saving time [34] which are not consistent with the findings of present study. This may be due to the different software and research community in these studies. Like the present study, the study of Lima et al. [37] showed that the computerized nursing process is useful in developing the nursing process, facilitating data collection, diagnostic reasoning, and identifying the clinical signs of newborn in neonatal units.

Most nurses have a positive idea on the use of the nursing process, but have little interest in using it because of it being time consuming [19]. The reduction in the time taken for completing the nursing documents, without decreasing the quality of collected data is an important advantage of information technology. This results in increased nursing time to stay with patients [48]. It should be noted that the time it takes to use a computerized system is inversely related to the resources of the information technology system [49]. So, using advanced software programs is a time management strategy that facilitates and accelerates the implementation of the nursing process because software programs allow nurses to enter assessment information quickly [19].

The growth of information technology and systems has been improving nursing care. Significantly. Using indicators of quality care, we find that information systems and software in nursing care help to organize and manage the increasing volume of information and data required by the nurse to develop their actions, saving time by recording the technical and scientific documents electronically. It also provides databases that can be used for research [48]. In this study, in addition to having the above features, we found that software that prioritizes problems based on the nursing assessment contributed to the positive results. Also using various valid and up to date nursing textbooks to develop the content of the software and finally the user friendly design of software had a direct impact on the student’s attitude towards using the software. “Fast implementation”, “data mining algorithms” and “forecasting techniques” are important features of new software and should be considered in the field of health care information technology [50, 51].

In the present study, the lowest level of students' attitudes towards the software was respectively related to the “feeling of fairness in labor division based on the software”, “the effectiveness of the software in determining your workload” and “the feeling of satisfaction in labor division based on the software”.

Regarding the cases with lowest level of student’s attitude towards the software, it should be noted that based on a review of extensive texts, in any of the studies issues such as observing the fairness of software-based division of work, the effectiveness of the software in determining workload and nursing students' satisfaction with software based work division has not been studied. However, it seems that since the clinical education system used in this study every student was responsible for all the care of the particular patient. The patients are transferred to the students based on the student’s academic knowledge and ability related to their semesters. However, proper division of tasks or assignment of student’s workload by their clinical instructors using the nursing process software has not been considered. So it is suggested that nurse educators evaluate students in terms of the amount of activities and clinical learning software data especially in the areas of diagnosis, nursing intervention and implementation.

Nursing students’ attitudes toward computers play a significant role in the successful implementation of information technology [52]. Education has an important effect on healthcare providers attitudes towards the nursing process [53–55]. Furthermore, nursing education has a major part to play in improving the use of the nursing process and thus promoting the overall quality of nursing care [56]. However, the lack of technology training during college courses, short and inadequate training time, lack of retraining and lack of tools [48] together with a Lack of reference information [57] are the main challenges for nurse education. Considering the tendency of today’s generation of nursing students to have a positive attitude toward technology and using the web supported learning methods will improve the quality of nursing education [58]. So in this study it was important to assess the nursing student’s attitude toward using the nursing process software as an educational tool for delivering patient care. Along with the numerous benefits of computer programs, some challenges of information technology were noted. Altering the routine care process and practices, deviating from direct patient care had a negative effect on decision making and patients outcome [59] that may impact on using information technology of nursing process. Other factors that may influence nurses’ attitudes toward computer software include the existence of a minimum set of nursing data and standard nursing terms in computer software [28]. It should be noted that in the current study, the Software’s comprehensiveness and the use of NANDA contribute to the validity of our findings. While the use of International Nursing Diagnosis and up-to-date content using standard nursing text books and applicability and simplicity of software contributed in removing the limitations of the information technology related to the nursing process. Therefore, according to our findings nursing managers and nursing education authorities should try to remove these existing barriers and provide the appropriate facilities to enhance the implementation of the computerized nursing process. Furthermore, the surest way to increase acceptance and positive attitudes, and thus ensure improvements in patient care, is to engage nurses as full stakeholders in implementing and improving these technologies [60].

In general, from the student’s response, the implementation of the computerized nursing process allows improved diagnosis accuracy, systematic and complete care and documentation. Although the implementation of a computer system is expensive and requires a great deal of planning and training, such systems can significantly improve patient safety by enhancing the quality of appropriate care [61].

In this study, female students had a significantly more positive attitude toward the nursing process software than male students. Also, the attitude scores of the younger students were significantly higher. In other studies a positive attitude of female nurses or nursing students toward the traditional nursing process is reported [14, 62]. It should be noted that in these studies, student’s attitudes towards computerized nursing process software have not been addressed, so this needs to be investigated in future studies. However, it appears that in most of the studies, including the current study, most of the study's participants were women, influenced by the traditional and feminine nature of the nursing profession and that this can have an effect on the results. Similar to this study, Singh and Masango [63] found that younger healthcare practitioners were more interested in using nursing information systems. But in the study of BabaMohamadi et al. [28], younger nurses had somewhat negative attitudes toward the impact of computerized nursing programs for patient care compared to older nurses. This may be affected by the low efficacy of one-step software used in this study for learning and providing care by young nursing students. It seems that nowadays younger students are more willing and satisfied using nursing process software than older students because of their familiarity with the world of technology and informatics and the greater attractiveness of such technologies. It is also likely that students who are more experienced in the professional and educational fields will be more aware of the disadvantages of establishing different care giving practices based on the computerized nursing process program.

### The advantages/disadvantage of this study

#### The advantages of this study

- Promoting the use of the nursing process in patient care.
- Improving the awareness and knowledge of nursing students about the nursing process.
- More accurate and more efficient implementation of the nursing process by clinical health care providers.
- Outline the need to establish computer-based equipment and infrastructure to implement the nursing process.

#### The disadvantage of this study

- Possibility of students relying on the software contents and functions.
- Possibility of reducing critical thinking and independent management of nursing students considering the relationship between the data in the different stages of the nursing process (patient assessment, nursing diagnoses, planning, implementation and evaluation) when using the nursing process software.

## Conclusions

Nursing students had a positive attitude about nursing process software, it seems that understanding the necessity of using the nursing process software in the age of information technology and also the efficiency of nursing process software on facilitating and accelerating the high quality care played a great role in creating the positive attitudes of nurses and nursing students. So this software can be used to improve the quality of care to the patients, using this software in the areas of teaching, research and clinical practice of students is also recommended. In this regard, changing policies both in nursing and in nurse education to develop, adapt and use the nursing process software is an important responsibility of the nursing authorities today. Also, providing educational and clinical technology equipment, periodic evaluation of software by stakeholders and promoting it and establishing training courses can be fundamental steps in operationalizing the findings of this research.

## Data Availability

The datasets used and/or analyzed during the current study are available from the corresponding author on reasonable request.

## Abbreviations

SPSS: Statistical Package for Social Sciences
ICU: Intensive care unit
